# Diethylenetriaminepentaacetic Acid‐based Conducting Solid Polymer Electrolytes Impede Lithium Dendrites and Impart Antioxidant Capacity in Lithium‐Ion Batteries

**DOI:** 10.1002/advs.202404506

**Published:** 2024-08-09

**Authors:** Yuli Zang, Muhammad Irfan, Zeheng Yang, Weixin Zhang

**Affiliations:** ^1^ School of Chemistry and Chemical Engineering Hefei University of Technology Hefei Anhui 230009 P. R. China; ^2^ Department of Chemical and Energy Engineering Pak‐Austria Fachhochschule: Institute of Applied Sciences and Technology Mang Haripur Pakistan

**Keywords:** electrochemical stability, high ionic conductivity, high voltage, ion‐polarized diethylenetriaminepentaacetic acid, lithium‐ion batteries

## Abstract

In the development of lithium‐ion batteries (LIBs), cheaper and safer solid polymer electrolytes are expected to replace combustible organic liquid electrolytes to meet the larger market demand. However, low ionic conductivity and inadequate cycling stability impede their commercial viability. Herein, a novel flexible conducting solid polymer electrolytes (CSPEs) based on polyvinyl alcohol (PVA) and ion‐polarized diethylenetriaminepentaacetic acid (P‐DETP) is developed for the first time and applied in LIBs. PVA and P‐DETP form a compact polymer network through hydrogen bonding, enhancing the thermomechanical stability of CSPE while restricting the migration of larger anions. Furthermore, density functional theory calculations confirm that P‐DETP can facilitate the dissociation of Li^+^‐TFSI^‐^ via electrostatic attraction, resulting in increased mobility of lithium ions. Additionally, P‐DETP contributes to the formation of a stable electrode‐electrolyte interface layer, effectively suppressing the growth of lithium dendrites and improving antioxidant capacity. These synergistic effects enable CSPE to exhibit remarkable properties including high ionic conductivity (2.8 × 10^−4^ S cm^−1^), elevated electrochemical potential (5.1 V), and excellent lithium transference number (0.869). Notably, the P‐DETP/LiTFSI CSPE demonstrates stable performance not only in LiFePO_4_ batteries but also adapts to high‐nickel ternary LiNi_0.88_Co_0.06_Mn_0.06_O_2_ cathode, highlighting its immense potential for application in high energy density LIBs.

## Graphical abstract

Ion‐polarized diethylenetriaminepentaacetic acid promotes the dissociation of lithium salts by electrostatic attraction and forms the network structure with polyvinyl alcohol by hydrogen bonding. The fast single lithium‐ion transport channel accelerates Li^+^ migration, which is conducive to the formation of stable solid electrolyte interfaces, inhibiting the growth of lithium dendrites, and achieving high cycling performance of lithium batteries.

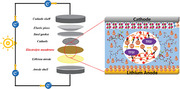

## 1 Introduction

At present, with the growing demand for portable consumer electronic devices, energy‐powered vehicles, large‐scale grid energy storage systems, and higher requirements are placed on the performances of lithium‐ion batteries (LIBs) such as energy density, safety, and cycle life.^[^ ^1^ ^]^ Lithium metal has the highest theoretical capacity (3860 mAh g^−1^) and the lowest electrochemical reduction potential (−3.04 V versus standard hydrogen electrode). It has long been considered a highly promising anode in the high energy density LIBs.^[^ ^2^ ^]^ However, the organic liquid electrolytes used in traditional commercial LIBs have poor thermal stability and high flammability, which may cause catastrophic battery fires or even explosions and other serious safety issues.^[^ ^3^ ^]^ Addressing the safety issue is critical for the commercial success of LIBs. The disadvantage of LIBs can be alleviated by using solid electrolytes (SSEs) instead of flammable organic liquid electrolytes.^[^ ^4^ ^]^

In the interior of all‐solid‐state lithium‐ion batteries (ASSLIBs), the solid‐state electrolytes (SSEs) act not only as separators but also as ionic conductors, which greatly affects the electrochemical performances of the battery. Generally, SSEs can be classified into two categories: inorganic solid electrolytes (ISEs) and solid polymer electrolytes (SPEs).^[^ ^5^ ^]^ ISEs usually consist of solid electrolytes of sulfides (such as Li_10_GeP_2_S_12_) and oxides (i.e., garnet, perovskite, NASICON or LiPON), which own excellent ionic conductivity, wide electrochemically stable window, and the ability to form a stable solid electrolyte interface (SEI) layer as an organic liquid electrolyte.^[^ ^6^ ^]^ However, ISEs exhibit high interface resistance and poor film processability (fragile), which seriously hinders their future practical application.^[^ ^7^ ^]^ In contrast, SPEs have excellent flexibility and satisfactory electrode compatibility. Thus, SPEs become a more suitable candidate material for ASSLIBs.^[^ ^8^ ^]^ However, SPEs have low ionic conductivity (< 10^−5^ S cm^−1^) and poor mechanical strength at room temperature, which is far below the actual standard.^[^ ^9^ ^]^ The construction of a crosslinking network is one of the effective methods to improve the conductivity of electrolytes. In addition, the cross‐linked network can promote relatively uniform lithium‐ion flux, adapt to the volume change of lithium deposition, and thus prevent dendrite growth.^[^ ^10^ ^]^

Poly (ethylene oxide) (PEO)/lithium salt polymer electrolyte was first proposed in the 1970s.^[^ ^11^ ^]^ However, its low ionic conductivity (< 10^−5^ S cm^−1^) hinders the transfer of Li^+^ between the electrolyte and the electrode. In addition, lithium‐ion transfer number (LTN) is also an important parameter to evaluate the electrolyte membrane performance.^[^ ^12^ ^]^ The high performance of the battery is always provided in an electrolyte with a higher LTN value (≈1) rather than a lower LTN value (such as LTN = 0.2), even if the latter has an order of magnitude higher ionic conductivity.^[^ ^13^ ^]^ The most direct way to achieve high LTN is to confine the anionic functional groups to a limited activity space to form conducting solid polymer electrolytes (CSPEs). However, CSPEs usually have limited ionic conductivity. It can be improved through the modification of anion chemistry,^[^ ^14^ ^]^ the design of nanostructure ion conduction channels,^[^ ^15^ ^]^ and the realization of customized polymer structures^[^ ^16^ ^]^ to achieve faster kinetics of Li ions. In addition, in order to provide highly reversible lithium metal stripping/plating and initial performance, CSPEs need to be able to form a stable solid electrolyte interface layer on the electrode.

The polymers including Polyvinylidene fluoride (PVDF) and PEO were commonly used for the preparation of solid polymer electrolytes but these polymers showed low mechanical and thermal stabilities. In contrast, polyvinyl alcohol (PVA) and brominated poly (2,6‐dimethyl‐1,4‐phenylene oxide) (BPPO) are more attractive due to their promising features.^[^ ^17^ ^]^ The PVA has a large number of hydroxyl groups on the carbon backbone, which has significant advantages over other polymers. The −OH groups act as subordinate functional groups and are excellent hydrogen bond donors, which have strong interactions with lithium ions and provide a path for lithium‐ion migration. Moreover, the −OH groups greatly increase the number of hydrogen bonds, which is conducive to enhancing the Li^+^ transport in CSPEs. In addition, the −OH groups are chemically active and can interact with other characteristic functional groups. PVA is also a plasticizer with excellent film‐forming properties. Its molding ability is better than that of other polymers, so the processing of PVA film is relatively simple and flexible.^[^ ^18^ ^]^ On the other hand, the BPPO has also some attractive features compared to PVDF and PEO. The BPPO has low cost, high thermal stability, extraordinary mechanical stability, and can be easily obtained by simple modification strategies. The addition of characteristic functional groups to BPPO polymers can also effectively increase the mobility of Li^+^ ions in CSPEs.^[^ ^19^ ^]^

Herein, strong hydrophilic PVA is selected as the polymer matrix, and ion‐polarized diethylenetriaminepentaacetic acid (P‐DETP), and quaternized brominated poly 2, 6‐dimethyl‐1,4‐phenyleneoxide (Q‐BPPO) are designed and synthesized. Novel functional groups (strong electron‐absorbing groups and hydrophobic structures) are introduced into the polymer matrix to improve the thermodynamic and electrochemical properties of CSPEs. The electrostatic attraction of the carboxylic group of P‐DETP will dissociate the lithium salts, releasing more freely moving lithium ions. The introduction of alkyl segments can inhibit the strong hydrophilicity of PVA. In addition, PVA and P‐DETP can form a dense polymer network structure through hydrogen bonding. This not only enhances the thermal stability and mechanical properties of the CSPEs but also greatly limits the free movement of anions. At the same time, the network structure provides a fast migration channel for lithium ions. It is beneficial to increase the mobility and ionic conductivity of Li^+^. In particular, theoretical calculations have verified that P‐DETP is conducive to the construction of stable SEI and cathode electrolyte interphase (CEI) so that the CSPE has good compatibility with the electrode. The density functional theory (DFT) was used to calculate the strong interaction between P‐DETP and Li^+^, as well as the lowest unoccupied molecular orbital (LUMO) and highest occupied molecular orbital (HOMO) of various components. The PDA‐F CSPE is not only cycles stably in the LiFePO_4_ batteries but also adapts to the high‐nickel ternary NCM cathode, thus exhibiting excellent cycle performance.

## 2 Results and Discussion

### 2.1 Mechanism of P‐DETP Improving Polymer Electrolyte Properties

**Figure 1:**
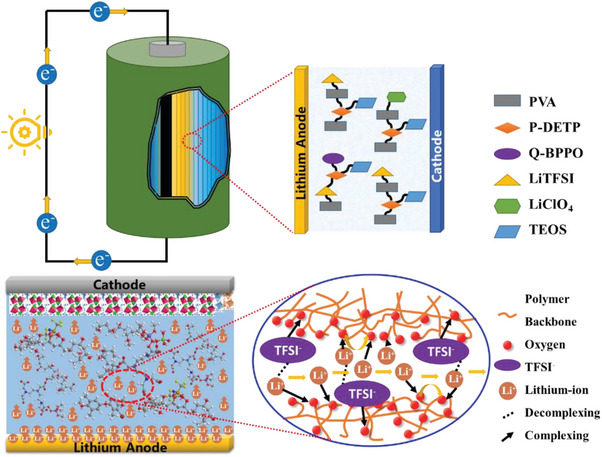
Schematic diagram of the CSPEs used in LIBs.

The application of the CSPEs in LIBs is shown in **Figure** **1**. The PVA matrix is rich in ‐OH groups, which provides excellent conditions for the formation of hydrogen bonds. The dense and flexible PVA cross‐linking network provides a fast and easy transport path for the migration of lithium ions. In addition, the macromolecular alkyl hydrophobic spacer group P‐DETP is rich in carboxyl groups, which can also provide binding sites for the migration of lithium ions, and promote the dissociation of lithium salts through hydrogen bonding and electrostatic attraction, thereby releasing more free lithium ions. Besides, due to the volume of the lithium salt anion group being much larger than that of lithium ions, its movement will be hindered by the cross‐linking network formed by PVA and P‐DETP, which can greatly reduce its interference in lithium‐ion transmission. These structures are designed to better improve the mobility of lithium ions and to improve ionic conductivity.

**Figure 2:**
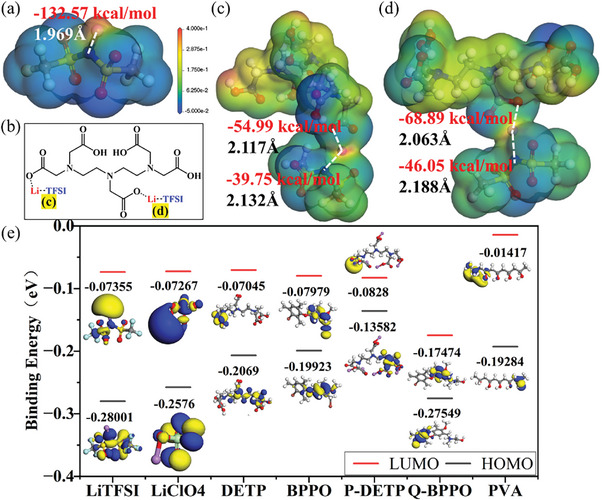
a) Binding energy and bond pitch of Li‐N bond in a single LiTFSI molecule. b) Interactions of P‐DETP and LiTFSI. c,d) Calculated binding energy and bond pitch between Li^+^ and TFSI^‐^ or different binding sites of P‐DETP. e) Calculated LUMO and HOMO of various components.

First‐principle calculations based on density functional theory (DFT) was used to calculate the strong interaction between P‐DETP and lithium salt (LiTFSI), as well as the lowest unoccupied molecular orbital (LUMO) and highest occupied molecular orbital (HOMO) of various components (**Figure** **2**). The geometry optimization and energy calculations were performed by Dmol3 program. As shown in Figure 2a, the binding energy between Li‐N in a single LiTFSI molecule is −132.57 kcal mol^−1^ with a bonding distance of 1.969 Å. The addition of P‐DETP can promote the dissociation of lithium salts. Each P‐DETP molecule has five carboxyl groups, which in different positions have a positive effect on the dissociation of LiTFSI (Figure 2b). The DFT results show that the Li‐O bond energy in P‐DFTP is stronger than the Li‐N bond energy in LiTFSI, confirming the carboxyl group of P‐DETP can increase the bond distance between Li^+^ and TFSI^‐^ and decrease the binding energy by electrostatic action. The carboxyl group located on the symmetry axis increases the bond distance of lithium‐ion and TFSI^‐^ anion to 2.132 Å and reduces the binding energy to −39.75 kcal mol^−1^ (Figure 2c). The strength of the four carboxyl groups on both sides is similar, so that the bond distance between Li^+^ and TFSI^‐^ is increased to 2.188 Å, and the binding energy is decreased to −46.05 kcal mol^−1^ (Figure 2d).

Since the composition of SEI and CEI is highly dependent on the LUMO and HOMO of the solvent, salt, or additive in the electrolyte, DFT calculations are performed to investigate the molecular orbital energies of the electrolyte components. As shown in Figure 2e, both P‐DETP and Q‐BPPO have low reduction potential. It contributes to the formation of a stable SEI layer, which can inhibit the side reactions of the lithium anode and electrolyte. Moreover, HOMO values of P‐DETP are higher than that of Q‐BPPO molecule, which means that P‐DETP prefers to decompose and participate in the CEI formation, which contributes to forming a stable CEI layer on the surface of the high‐voltage cathode, improving the oxidation resistance of the electrolytes and the cycle life of the batteries. Therefore, the P‐DETP is beneficial to construct stable SEI and CEI, thus effectively improving the electrolyte compatibility both with the lithium metal anode and high‐voltage cathode simultaneously.^[^ ^1^, ^8^ ^]^

### 2.2 Structure and Morphology Analysis of CSPEs

**Figure 3:**
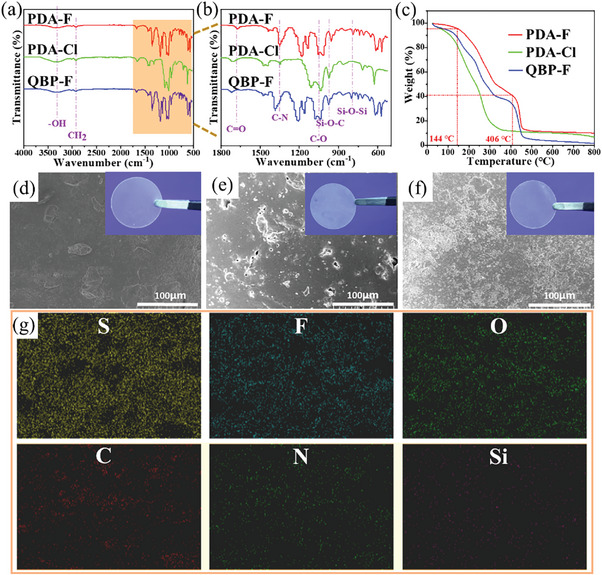
a,b) FTIR spectra of PDA‐F, PDA‐Cl, and QBP‐F CSPEs. c) TGA thermograms of the CSPEs. The surface SEM images of d) PDA‐F, (e) PDA‐Cl, and f) QBP‐F CSPEs. Inserts are the optical photographs of CSPEs. g) EDS map of S, F, O, C, N, and Si for PDA‐F CSPE.

CSPEs were prepared by blending P‐DETP and Q‐BPPO with PVA solution by the one‐pot method. FTIR testing of CSPEs was performed to analyze the structure of synthetic materials (**Figure** **3**a,b). The absorption peaks at ≈800 cm^−1^ correspond to the absorption peaks of Si─O─Si bonds, while the formation of Si─O─C resulted in a peak at 954 cm^−1^, which verifies the successful bonding of TEOS to the polymer segment.^[^ ^20^ ^]^ The strong absorption peak at 1050 cm^−1^ manifests the vibration of the primary alcohol C─O bond, while the ─C─N stretching vibration absorption in P‐DETP is located at 1350 cm^−1^.^[^ ^21^ ^]^ The absorption peak at 1715 cm^−1^ is assigned to the carbonyl (C═O) structure of the carboxylic acid in the synthesized CSPEs.^[^ ^22^ ^]^ The wide absorption peak at 3420 cm^−1^ indicates hydroxyl groups.^[^ ^21^ ^]^ The various characteristic absorption peaks as mentioned above validate the reactions between P‐DETP, TEOS, Q‐BPPO, and PVA, thereby confirming the successful introduction of various functional groups into the polymer matrix.

The introduction of various functional groups is conducive to the formation of amorphous morphology of the electrolyte film (Figure S2a, Supporting Information), resulting in a serious decrease in polymer crystallinity. Studies have shown that the (101) crystal plane of PVA corresponds to the sharp peak at 2θ = 19.6° in the XRD pattern.^[^ ^23^ ^]^ In Figure S2a, Supporting Information, the peak intensity of PDA‐F is significantly reduced, suggesting the amorphous structure, which is due to the good interaction between LiTFSI with P‐DETP and PVA soft long chains. The low crystallinity of the polymer electrolyte is conducive to enhancing the movement of the chain segment, thus speeding up the ion conduction.

To ensure that CSPEs can inhibit lithium dendrites during battery assembly and cycling, their mechanical properties were verified.^[^ ^24^ ^]^ The stress‐strain curve obtained by the tensile test is shown in Figure S2b, Supporting Information, and the results show the difference in the mechanical properties of CSPEs. The tensile strength of PDA‐F, PDA‐Cl, and QBP‐F are respectively 1.95, 1.28, and 1.16 MPa. Their fracture nominal strains are 102.1%, 84.9%, and 79.4%. From the perspective of lithium salt type, compared with LiTFSI, LiCiO_4_ significantly increases the fracture stress of CSPEs. From the DFT calculation results, the reduction potential of LiClO_4_ is lower than that of LiTFSI, which indicates that the LiTFSI will preferentially decompose and participate in the formation of SEI with rich LiF‐Li_3_N,^[^ ^1^, ^10^, ^13^ ^]^ thus effectively suppressing side reactions between Li anode and CSPEs. The comparison results of different macromolecular spacer groups show that Q‐DETD has a stronger ability to improve the mechanical properties of electrolyte membranes than Q‐BPPO.

To verify the safety of the batteries during the charging and discharging processes, the thermal stability of CSPEs was examined (Figure 3c, Figure S2c,d, Supporting Information). The melting temperature of the electrolyte film was determined through differential scanning calorimeter (DSC) tests at 30–200 °C. As depicted in Figure S2c, Supporting Information, PDA‐Cl exhibits a peak heat absorption at 90 °C, while PDA‐F and QBP‐F demonstrate melting points of ≈110 °C. The superior thermal stability of the former can be attributed to the dense network structure formed by abundant hydrogen bonds between its molecules, whereas the latter is due to the incorporation of polyphenylene ether structures. This results in an increased energy requirement for structural destruction, thereby exhibiting commendable thermal stability. Furthermore, thermogravimetric analysis (TGA) was performed on CSPEs which revealed that they initially experienced slight weight loss primarily due to water evaporation as a result of varying degrees of water absorption from air (Figure 3c, Figure S2d, Supporting Information). Subsequently, with increasing temperature, mass continues to decrease attributable to residual DMSO volatilization. PDA‐Cl reaches a decomposition temperature at ≈225 °C and undergoes significant weight loss; QBP‐F begins decomposing at ≈375 °C; and PDA‐F exhibits a decomposition temperature reaching ≈406 °C. This attractive thermal stability of PDA‐F is due to the linkage of hydrogen bond interactions between amine‐based macropolymers and PVA chains.^[^ ^25^ ^]^ Hydrogen bonding makes the structure of the polymer network denser and stronger and effectively improves the thermal stability and mechanical properties of the film.

The introduction of hydrophobic structure on polymer molecular chain can improve the mechanical properties of electrolyte membrane appropriately.^[^ ^19a,b^ ^]^ Water contact angle measurements were performed on synthetic CSPEs to study their hydrophobicity (Figure S2e–g, Supporting Information). The contact angle of PDA‐F, PDA‐Cl, and QBP‐F are respectively 46°, 37°, and 61°, indicating that the addition of P‐DETP and Q‐BPPO could improve the hydrophobicity of the membranes. This may be due to the interaction of the carboxyl group of P‐DETP or the benzene ring structure in Q‐BPPO with the electrolyte matrix. In addition, the action of macromolecular alkyl spacers and LiTFSI can also improve the hydrophobicity of CSPE compared to LiClO_4_. The improvement of the hydrophobicity of the electrolyte membrane can reduce the influence of ambient air to a certain extent, which is conducive to enhancing the stability of battery performance.

The smooth surface of the membrane facilitates the formation of a better SEI between it and the electrodes. The SEM scanning of CSPEs (Figure 3d–f, Figure S3, Supporting Information) shows that the electrolyte membrane surface of PDA‐F is relatively more uniform and smoother, and the internal structure is also denser, which is conducive to good interface contact stability and rapid ion transport. The surface of QBP‐F is not smooth, with many uneven protrusions. Moreover, QBP‐F presents a loose porous structure inside, which can make the interface contact performance poor, and adversely affect the battery performance. The surface of PDA‐Cl is rough and non‐uniform, with obvious holes and cracks (Figure S3e, Supporting Information). These forms are very unfavorable to the formation of a suitable SEI between the electrolyte film and the lithium anode, resulting in the worst electrochemical performance of its batteries. Compositional analysis of PDA‐F (C, O, F, S, N, Si) was performed using energy‐dispersive X‐ray spectroscopy (EDS) (Figure 3g, Figure S4, Supporting Information). All the major elements are evenly distributed on the electrolyte membrane (Figure 3g). Peaks of sulphur and silicon were observed at about 2 keV binding energies and nitrogen peaks at about 0.5 keV binding energies due to sulfonate and carboxylic acid anionic central groups, TEOS, and quaternary ammonium anionic central groups (Figure S4b, Supporting Information). These elements were present in PDA‐F, and their uniform distribution (Figure 3g) validates that P‐DETP, TEOS, and TFSI^‐^ were successfully combined with PVA substrates into the synthesized CSPEs. The hydrogen bond and electrostatic attraction interaction between various functional groups also have positive effects on the electrochemical performance of the electrolyte membrane. In addition, the thickness of the electrolyte membrane has a great influence on the energy density and internal resistance of the battery. Cross‐sectional SEM shows that the thickness of PDA‐F (80 µm) is thinner than that of PDA‐Cl (103 µm) and QBD‐F (220 µm), which is conducive to its excellent performance in batteries.

### 2.3 Electrochemical Properties and Interfacial Stability of CSPEs

**Figure 4:**
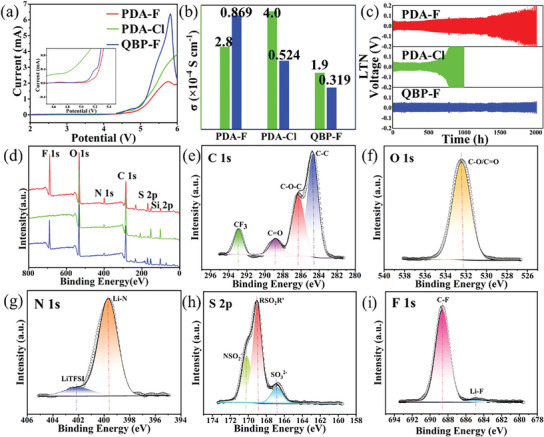
a) LSV curves of PDA‐F, PDA‐Cl, and QBP‐F CSPEs. b) ionic conductivities and LTNs of PDA‐F, PDA‐Cl, and QBP‐F CSPEs. c) Voltage profiles of Li/CSPEs/Li cells at a current density of 0.1 mA cm^−2^. (d) XPS wide scan spectra of PDA‐F, PDA‐C1, and QBP‐F CSPEs. e–i) The high‐resolution C 1s, O 1s, N 1s, S 2p, and F 1s of PDA‐F.

The CSPEs were subjected to linear scanning voltammetry (LSV) testing to verify their electrochemical stability window (**Figure** **4**a). The LSV of PDA‐F is up to 5.1 V, while the LSV of PDA‐Cl and QBP‐F are respectively 4.8 and 5.0 V, indicating that PDA‐F and QBP‐F have high electrochemical oxidation resistance and can be adapted to high‐voltage NCM ternary cathodes. To further characterize the oxidation stability of CSPEs in batteries, NCM8866/Li batteries were assembled with NCM8866 electrodes. The measured leakage current can directly evaluate the actual oxidation stability of the electrolyte. The results are shown in Figure S5a, Supporting Information. As can be seen from Figure S5a, Supporting Information, the leakage current of PDA‐F is small and stable when it is below 5.1 V, and increases sharply when it is 5.2 V, indicating that its electrochemical window is ≈5.1 V, which fully proves its excellent stability to high voltage cathodes. PDA‐F exhibits an excellent electrochemical stability window since the macromolecular structure of P‐DETP restricts the free migration of anions, resulting in a low concentration of anions on the cathode and promoting the formation of the SEI layer, thereby increasing electrochemical stability, and also enhancing the rapid conduction of lithium ions. Biphenyls from Q‐BPPO in QBP‐F cause higher electrochemical stability. Its strong ability to absorb electrons can further reduce the highest molecular energy level occupied by molecules (Figure 2e). These groups are difficult to oxidize, which increases the electrochemical stability window of the QBP‐F. It can be seen that the central structure of Q‐BPPO is very stable, which can greatly improve the oxidation resistance of the CSPE, so it is conducive to improving the cycle life of the batteries.

Ionic transport capacity is a key parameter to evaluate the battery performance, mainly including the determination of ion conductivity and LTN (Figure 4b). PDA‐F (2.8 × 10^−4^ S cm^−1^) and PDA‐Cl (4.0 × 10^−4^ S cm^−1^) have higher ionic conductivity than QBP‐F (1.9 × 10^−4^ S cm^−1^) at room temperature. P‐DETP provides more charge carriers within the polymer matrix to increase ionic mobility and the amorphous shape of the electrolyte membrane. Therefore, its addition has a beneficial effect on the improvement of ionic conductivity. Through the data processing results, it can be seen that ClO_4_^‐^ is more effective than TFSI^‐^ in increasing the ionic conductivity of batteries. It may be because the former is less electronegative, which is beneficial to the dissociation and migration of Li^+^. Furthermore, due to the macromolecular side chain group being fixed on the polymer matrix, the anion activity is limited to a certain extent, which gives the cation more space to move freely. The carboxylic and quaternary amine groups are the main functional groups that could play a crucial part in the battery working process. They improved the affinity of CSPEs for polar solvents, reduced the crystallinity of CSPEs, promoted the ionization of lithium ions and lithium salt anions, enhanced the ion transport of CSPEs, and made positive contributions to the improvement of ionic conductive properties. Furthermore, the addition of TEOS not only provides several hydrogen bonds for CSPEs but also builds the Si‐O‐Si network with flexible segmental motion, which is beneficial to improving ionic conductivity. Hence, the incorporation of TEOS and P‐DETP inside the PVA matrix has a positive contribution to improving ionic conductivity properties.

Due to the strong hydrogen bond between P‐DETP macromolecular groups and the PVA matrix, which forms a dense network structure, the activity range of anions is limited. This gives Li^+^ more room to roam. The carboxyl group of P‐DETP promotes the dissociation of lithium salts, reduces the binding energy of lithium ions and anions (Figure 2c,d), and greatly increase the number of freely migrated lithium ions. As a result, the PDA‐F obtained an excellent lithium‐ion migration number of 0.869, which laid a foundation for the good electrochemical performance of the battery. The LTN of PDA‐Cl is slightly worse at 0.524, and the LTN of QBP‐F is only 0.319. This further proves that the addition of P‐DETP has made a positive contribution to the electrochemical performance of the batteries.

The electrochemical stability of the cells assembled with CSPEs was studied by cyclic voltammetry (CV) (Figure S5b–d, Supporting Information). The shapes of the CV curves are similar, indicating that P‐DETP, Q‐BPPO, and TEOS do not undergo the electrochemical reaction in the voltage range of +2.5–4.2 V (versus Li/ Li^+^). It can be seen that their reversible redox peaks are mainly formed by the intercalation and separation of Li^+^ ions in the electrode.

The excellent ion conduction ability lays a good foundation for the long cycle life of batteries. At the same time, it is also necessary to pay attention to the interface stability of the membrane during the battery operation. To verify the ability of CSPEs to inhibit the lithium dendrite growth, we assembled Li/Li symmetric cells and tested their performance. During periodic constant current charging/discharging process, we evaluated the interface compatibility between CSPEs and lithium metal anode by using the specific capacity‐voltage curve (Figure 4c). The results showed that the symmetrical Li/Li batteries assembled by PDA‐F and QBP‐F as electrolyte membrane could undergo 2000 h cycle during the charge and discharge process of constant current density (0.1 mA cm^−2^) at room temperature, which is significantly better than that of PDA‐Cl. It is explained that the symmetrical batteries have good cycling performance, indicating that the reversibility of lithium intercalation/deintercalation is highly stable during battery operation. It is proved that the electrolyte membranes have excellent resistance to lithium dendrite growth, thus providing excellent interfacial contact between the electrolyte membranes and the lithium anodes and preventing short‐circuit phenomena. This facilitates the long‐cycle stability of the button batteries.

The formation and growth of lithium dendrites are mainly determined by the migration of lithium in the electrolyte membranes. Under unrestricted conditions, the random free migration of anions and cations within the electrolyte may lead to uneven deposition of Li on the surface of the Li anode, leading to the development of lithium dendrites and even battery short circuits. The introduction of P‐DETP in electrolyte can not only produce hydrogen bonds with PVA matrix, and enhance polymer network structure, but also dissociate lithium ions from lithium salt anions through electrostatic attraction, release more free lithium ions, and limit the movement space of lithium salt anions. Therefore, Li^+^ can migrate more smoothly and quickly in the electrolyte, and be uniformly embedded and deembedded on the Li anode, thus forming a wonderful SEI layer and inhibiting dendrite nucleation. XPS analysis was used to further identify the reaction products on the surface of the circulating Li anode corresponding to the electrolyte (Figure 4d–i, Figure S6, Supporting Information). Full spectrum and narrow area scanning of CSPEs demonstrated the presence of predictable elements C 1s, O 1s, N 1s, F 1s, S 2p and Si 2p (Figure 4d). The high‐resolution scans of XPS are deconvoluted to identify the chemical state of the elements. The CSPEs have a macromolecular carbon chain as the organic skeleton, which contains a large number of ether‐oxygen bonds (Figure 4e,f, Figure S6a,b,g–h, Supporting Information). The oxygen (O) atoms provide sufficient coordination bonds for Li and favorable conditions for ion transport. In addition, ether bonds have a higher dielectric constant (ε≈5), which can promote the dissociation of ion pairs in Li salts and release more free lithium conduction.^[^ ^26^ ^]^ The amine group has a strong interaction with TFSI^‐^ anions in lithium salts (Figure 4g–h, Figure S6i,j, Supporting Information), which can significantly reduce the mobility of TFSI^‐^, thereby increasing the lithium‐ion mobility and lithium transfer number of the single‐ion conductor electrolyte.^[^ ^27^ ^]^ The introduction of fluorine into the electrolyte can effectively stabilize its interface contact with the lithium metal anode, thereby improving battery performance. Fluorinated segments and lithium salts in polymers can provide a source for the formation of LiF‐rich components at the SEI, also improving battery performance (Figure 4i).^[^ ^28^ ^]^

### 2.4 Battery Performances of CSPEs

**Figure 5:**
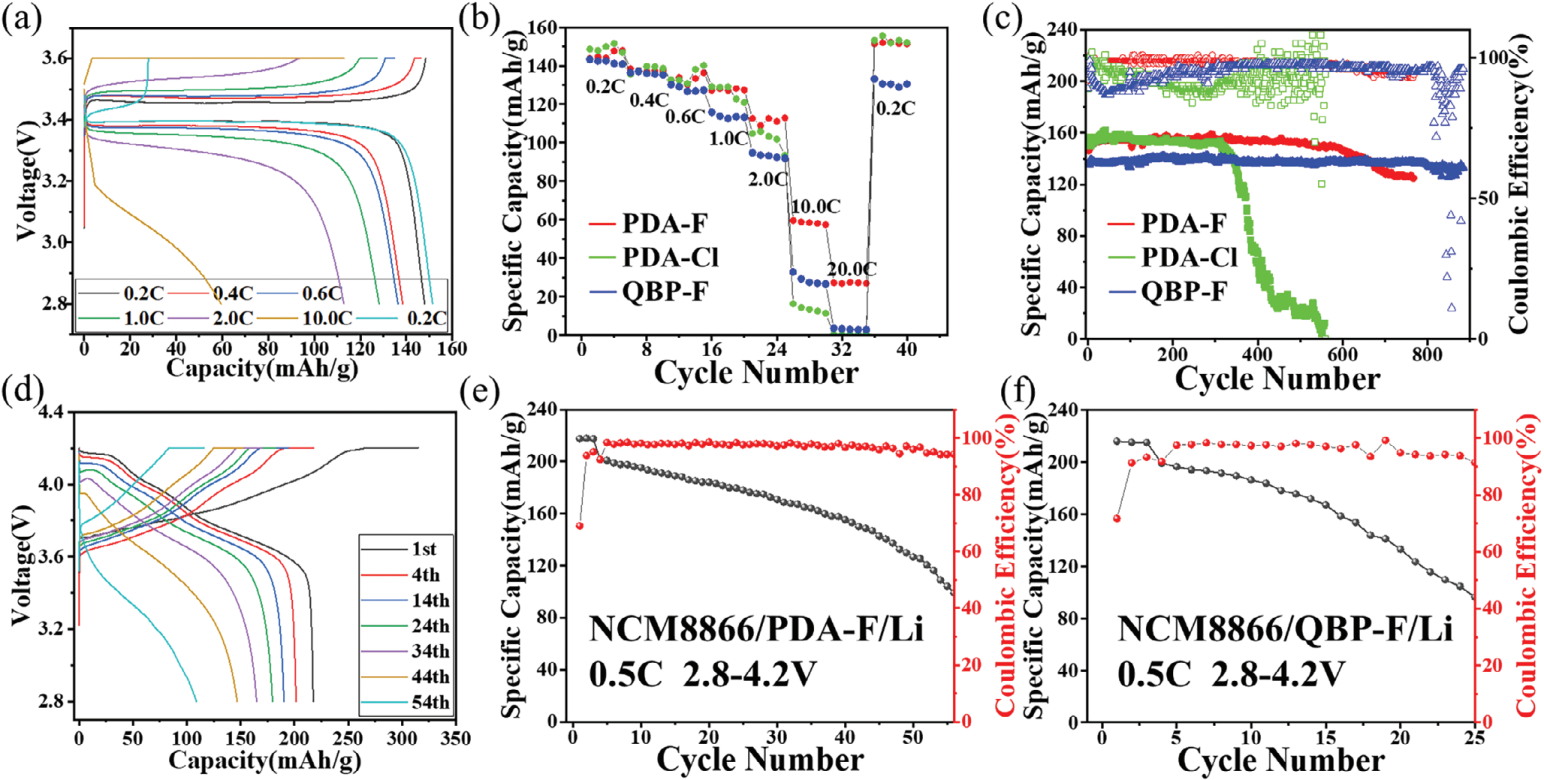
a) Galvanostatic charge/discharge profiles of the LFP/PDA‐F/Li cell at 0.2–10 C rates. b) The rate capability of LFP/CSPEs/Li cells for PDA‐F, PDA‐Cl, and QBP‐F CSPEs at 0.2–10 C rates. c) Discharge capacity and coulombic efficiency of LFP/CSPEs/Li cells at 0.3 C and 25 °C. d) Voltage plateaus of the 1st, 4th, 14th, 24th, 34th, 44th, and 54th cycles of the NCM8866/PDA‐F/Li battery at 0.3 C and 25 °C. Discharge capacity and coulombic efficiency of e) NCM8866/PDA‐F/Li cells, and f) NCM8866/QBP‐F/Li cells at 0.5 C and 25 °C.

To verify the feasibility of CSPEs applications in LIBs, coin batteries were assembled by combining CSPEs with LFP cathodes and lithium anodes. The rate performance of the batteries is first verified, and it performs well at a small rate (Figure S7a,b, Supporting Information). The test results show that the discharge‐specific capacity of PDA‐Cl and QBP‐F under low‐rate conditions is not much different from that of PDA‐F, but it drops significantly at high‐rate (20 C) (**Figure** **5**a,b). It is because the poor ionic migration number of PDA‐Cl and QBP‐F leads to increased concentration polarization at high current densities, which accelerates battery failure. PDA‐F significantly outperforms other products in continuous large C‐rate cycles. And the batteries have a relatively stable charge/discharge capacity in cycles of different rates. The LFP/PDA‐F/Li cell delivers outstanding rate performance with discharge capacities of 147, 137, 133, 127, 112, 59, 27, and 151 mAh g^−1^ at rates of 0.2, 0.4, 0.6, 1.0, 2.0, 10.0, 20.0, and 0.2 C, respectively (Figure 5b). The PDA‐F electrolyte membrane is more suitable for LIBs and can play a better role in the working process of the batteries. It has been demonstrated in the long cycle life test of the batteries.

The battery performance of LiFePO_4_/CSPEs/Li with PDA‐F, PDA‐Cl, and QBP‐F CSPEs at 0.3 C and 25 °C was investigated (Figure 5c). The long cycle performance results demonstrate that the discharge capacity of PDA‐F is better than that of the others. The battery of PDA‐F with P‐DETP shows excellent discharge‐specific capacity of up to 145 mAh g^−1^ at 0.3 C. After 600 cycles, the capacity retention rate reached 98%, and the coulomb efficiency is always close to 100% (Figure S7c, Supporting Information, Figure 5c). The electrochemical performance of PDA‐F cells is superior to that of other recently reported CSPEs listed in Table S2, Supporting Information. The discharge capacity of the QBP‐F with Q‐BPPO is only about 140 mAh g^−1^, but it can maintain a stable long cycle life of up to more than 800 cycles. Compared with them, the PDA‐Cl battery has poor performance (Figure 5c) since the LiClO_4_ lithium salt structure is unstable and cannot inhibit the growth of lithium dendrites. Considering these observations, it can be seen that P‐DETP and Q‐BPPO have their unique advantages, and P‐DETP has better performance.

To better verify the outstanding conductivity and stability of CSPEs, NCM8866/PDA‐F/Li and NCM8866/QBP‐F/Li cells were fabricated and the cycle performance tests were carried out between 2.8 and 4.2 V at 25 °C and 0.5 C. Unfortunately, the PDA‐Cl membrane has poor performance and is hard to assemble for the fabrication of the battery during cycling. The first three cycles are run at a lower rate of 0.2 C to make the batteries run better. PDA‐F's discharge capacity at 0.2 C is 217 mAh g^−1^, and its initial discharge capacity at 0.5 C is 200 mAh g^−1^ with a coulombic efficiency of ≈98%. After 50 cycles at 0.5 C, the discharge capacity (Figure 5d,e) is retained at 63%. QBP‐F's discharge capacity at 0.2 C is 215 mAh g^−1^, and its initial discharge capacity at 0.5 C is 196 mAh g^−1^ with a coulombic efficiency of ≈97%. After 21 cycles at 0.5 C, the discharge capacity (Figure 5f) is retained at 63%. Based on the above research, we further assembled NCM9055/Li cells to evaluate the performance and application of PDA‐F and QBD‐F electrolytes. The NCM9055/PDA‐F/Li cell delivers outstanding rate performance with discharge capacities of 226.12, 214.27, 177.41, 167.99, and 156.13 mAh g^−1^ at rates of 0.1, 0.3, 0.5, 0.7, and 1.0 C (Figure S9a, Supporting Information). However, the rate performance of the NCM9055/QBD‐F/Li cell is significantly worse. The NCM9055/PDA‐F/Li cell is tested for cycling performance in a voltage range of 2.5–4.3 V (Figure S9b,c, Supporting Information). Its initial discharge capacity at 0.5 C is 167.94 mAh g^−1^. After 31 cycles at 0.5 C, the discharge capacity is 137.66 mAh g^−1^ and the capacity retention rate is 81.97%. The coulombic efficiency of the battery in the working process is maintained at about 90%. The dense polymer network structure in PDA‐F makes the Li^+^ transport path more rapid and convenient and provides a prerequisite for good cycle performance of the batteries. In addition, the stable electrolyte‐electrode interface layer improves the mechanical properties of the electrolyte film, inhibits the nucleation and growth of Li dendrites well, and is conducive to the long cycle life of the battery. Besides, P‐DETP introduces strong electron‐absorbing groups into the electrolyte membrane, and the addition of fluorinated LiTFSI contributes to the formation of LiF which is the important active component of the stable interface layer, and the salt‐rich system also has the potential to stabilize the electrolyte under high voltage conditions. The rational application of these strategies is helpful in broadening the electrochemical stability window of the batteries. Furthermore, the wide electrochemical stability window greatly improves the ability of electrolyte to withstand high voltage and resist decomposition, so that the battery can adapt to the high voltage cathode and stable circulation. It is further proved that PDA‐F membrane has excellent high‐voltage resistance.

To better understand and analyze the variation of internal resistance during the charging and discharging process of the batteries, the charge transfer resistance (R_ct_) of the LFP/Li batteries and the NCM8866/Li batteries were tested separately (Figure S8, Supporting Information). The Nyquist impedance plots of cells after the third and twentieth cycles at 0.5 C consists of two semicircles and a diachronic line. After three cycles, the first smaller semicircle in the high‐frequency region is attributed to the SEI layer resistance. The SEI resistance of the LFP/Li battery sample with PDA‐F electrolyte (51.84 Ω) (Figure S8a, Supporting Information) is relatively lower than that of the sample with PDA‐Cl electrolyte (66.6 Ω) and QBD‐F (60.73 Ω). The semicircle in the mid‐frequency region represents the charge transfer resistance. Among the three, the R_ct_ of LFP/PDA‐F/Li increases only to 97.9 Ω, with the smallest internal resistance change (Figure S8a, Supporting Information). In contrast, the R_ct_ of NCM8866/PDA‐F/Li increases from 56.77 to 375.3 Ω, and even the R_ct_ of NCM8866/QBD‐F/Li increases sharply from 68.83 to 520.4 Ω (Figure S8b, Supporting Information). The results indicate that the internal resistance of PDA‐F in LFP cells is significantly lower than that in NCM8866 cells. The minimal change in internal resistance of PDA‐F in LFP/Li batteries suggests excellent interface stability of the electrolyte, thereby providing favorable conditions for the development of cyclically stable LIBs. Conversely, NCM8866/PDA‐F/Li exhibits high internal resistance and poor cycle stability, suggesting inadequate electrolyte interface and consequent reduction in battery capacity.

**Figure 6:**
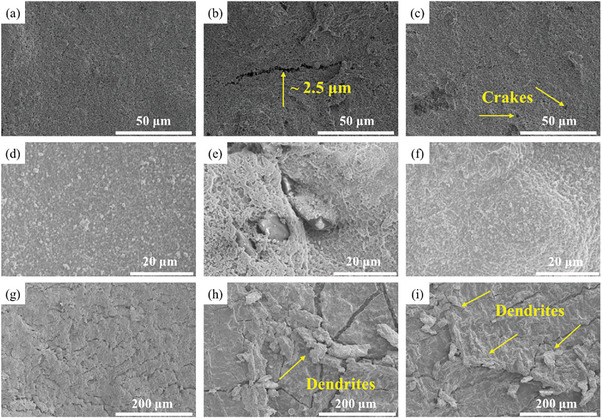
The SEM micrographs of NCM8866 after cycling from a) NCM8866/PDA‐F/Li cell, b) NCM8866/PDA‐Cl/Li cell, and c) NCM8866/QBP‐F/Li cell. The SEMs micrographs of the surface between CSPEs and Li anode after cycling from d) NCM8866/PDA‐F/Li cell, e) NCM8866/PDA‐Cl/Li cell, and f) NCM8866/QBP‐F/Li cell. The SEMs micrographs of Li surface after cycling from g) NCM8866/PDA‐F/Li cell, h) NCM8866/PDA‐Cl/Li cell, and i) NCM8866/QBP‐F/Li cell.

In order to better observe the changes of CSPEs during batteries cycling and the influences on the cathodes and anodes, and failure analysis was carried out (**Figure** **6**). The assembled NCM8866/CSPEs/Li batteries are disassembled after cycling 10 times at 0.5 C, when they are full discharge. The cathode sheet of NCM8866/PDA‐F/Li battery has almost no change, and the surface is flat and uniform (Figure 6a). The cathode of NCM8866/PDA‐Cl/Li cell is poor, with obvious cracks about 2.5 microns wide (Figure 6b). Whereas, the cathode of NCM8866/QBP‐F/Li battery has some smaller holes on the surface (Figure 6c). The surface of the cycled PDA‐F is relatively smooth and there are no obvious defects. The formation of the dense SEI layer can not only inhibit the growth of lithium dendrites but also protect the electrolyte membrane and reduce its loss (Figure 6d). The properties of the PDA‐Cl film are poor, so the surface is very uneven after circulation, with obvious defects (Figure 6e). Although SEI is also generated on the surface of QBP‐F, it is uneven, which makes the cycling performance stability of the battery poor (Figure 6f). Observing the surface of lithium anodes, it can be seen that the surface of the lithium sheet in NCM8866/PDA‐F/Li battery is smooth, and there is no obvious lithium dendrite and cracking (Figure 6g). Meanwhile, wide cracks and irregular dendrites occur on the surface of the lithium anode in NCM8866/PDA‐Cl/Li battery (Figure 6h). The surface of the anode in NCM8866/QBP‐F/Li cell also has slight dendrite and crack formation, making the surface worse compared to the Li toward PDA‐F (Figure 6i). In conclusion, it is further proved that the introduction of P‐DETP and the selection of LiTFSI promote the formation of excellent SEI layers. This makes PDA‐F have good interfacial stability with the cathode and Li anode during battery cycling, thereby improving the cycle stability and long cycle life of the battery.

## 3 Conclusion

In conclusion, we used the blending strategy to prepare the synthesized P‐DETP and PVA matrix into CSPEs, which were successfully applied it to LIBs. P‐DETP and PVA construct a polymer network through hydrogen bonding, which reduces the crystallinity of CSPEs and improves the mechanical properties and thermal stability. The dense network structure limits the migration of large anions and provides a convenient pathway for the conduction of lithium ions. In addition, P‐DETP can dissociate lithium salts through electrostatic attraction, release more freely moving lithium ions, and make the CSPEs obtain 2.8 × 10^−4^ S cm^−1^ ionic conductivity and 0.869 lithium‐ion migration number. The introduction of P‐DETP is also beneficial to the construction of CEI and SEI, inhibits the occurrence of interface side reactions, and improves the ability of CSPEs to withstand high voltage decomposition (5.1 V). The good interface compatibility between electrolyte and electrode makes the polarization voltage output of PDA‐F CSPE reach 2000 h at 0.1 mA cm^−2^. The LFP/PDA‐F/Li battery has a discharge capacity of up to 145 mAh g^−1^ at 0.3 C and 25 °C. After 600 cycles, the capacity retention rate is as high as 98%. The NCM8866/PDA‐F/Li battery can discharge up to 200 mAh g^−1^ at 0.5 C, and the coulomb efficiency can reach 95%. PDA‐F CSPE has the advantages of low cost, simple preparation process and easy operation, and has a good commercial prospect in the development of high energy density lithium‐ion battery applications.

## Conflict of Interest

The authors declare no conflict of interest.

## Supporting information

Supporting Information

## Data Availability

The data that support the findings of this study are available from the corresponding author upon reasonable request.
