# Supplementary material for: Diethylenetriaminepentaacetic Acid‐based Conducting Solid Polymer Electrolytes Impede Lithium Dendrites and Impart Antioxidant Capacity in Lithium‐Ion Batteries
Source: Adv Sci (Weinh). 2024 Aug 9;11(38):2404506. doi: 10.1002/advs.202404506 (PMC11481259; doi:10.1002/advs.202404506)
Supplement: Supplementary file 1 — Supporting Information [file ADVS-11-2404506-s001.docx]

Supporting Information

Diethylenetriaminepentaacetic acid-based conducting solid polymer electrolytes impede lithium dendrites and impart antioxidant capacity in lithium-ion batteries

*Yuli Zang, Muhammad Irfan,* Zeheng Yang, and Weixin Zhang**

Yuli Zang, Zeheng Yang, Weixin Zhang

School of Chemistry and Chemical Engineering, and School of Materials Science & Engineering, Hefei University of Technology, Hefei, Anhui, 230009, PR China

Email: wxzhang@hfut.edu.cn

Muhammad Irfan
Department of Chemical and Energy Engineering, Pak-Austria Fachhochschule: Institute of Applied Sciences and Technology, Mang, Haripur, Pakistan

Email: muhammad.irfan@fcm3.paf-iast.edu.pk

1. Experimental Section

1.1. Materials

Diethylenetriaminepentaacetic acid, dimethylacetamide, lithium hydroxide, BPPO, and PVA were purchased from Aladdin. LiFePO_4_, acetylene black, polyvinylidene fluoride (PVDF), tetraethyl orthosilicate (TEOS), N-N, dimethylacetamide (DMAc), dimethyl sulfoxide (DMSO), dimethyl sulfoxide, lithium bis(trifluoromethanesulfonyl)imide, dimethylethanolamine, toluene, lithium perchlorate, ethyl acetate, N-Methyl-2-pyrrolidone (NMP), and diethyl ether were purchased from Sinopharm Chemical Reagent Co., Ltd. LiNi_0.88_Co_0.06_Mn_0.06_O_2_ (NCM8866) and LiNi_0.90_Co_0.05_Mn_0.05_O_2_ (NCM9055) were purchased from Gotion High-tech Co., Ltd. These chemicals possess the quality of analytical grade and were applied without further purification.

1.2. Preparation of Solid Polymer Electrolytes


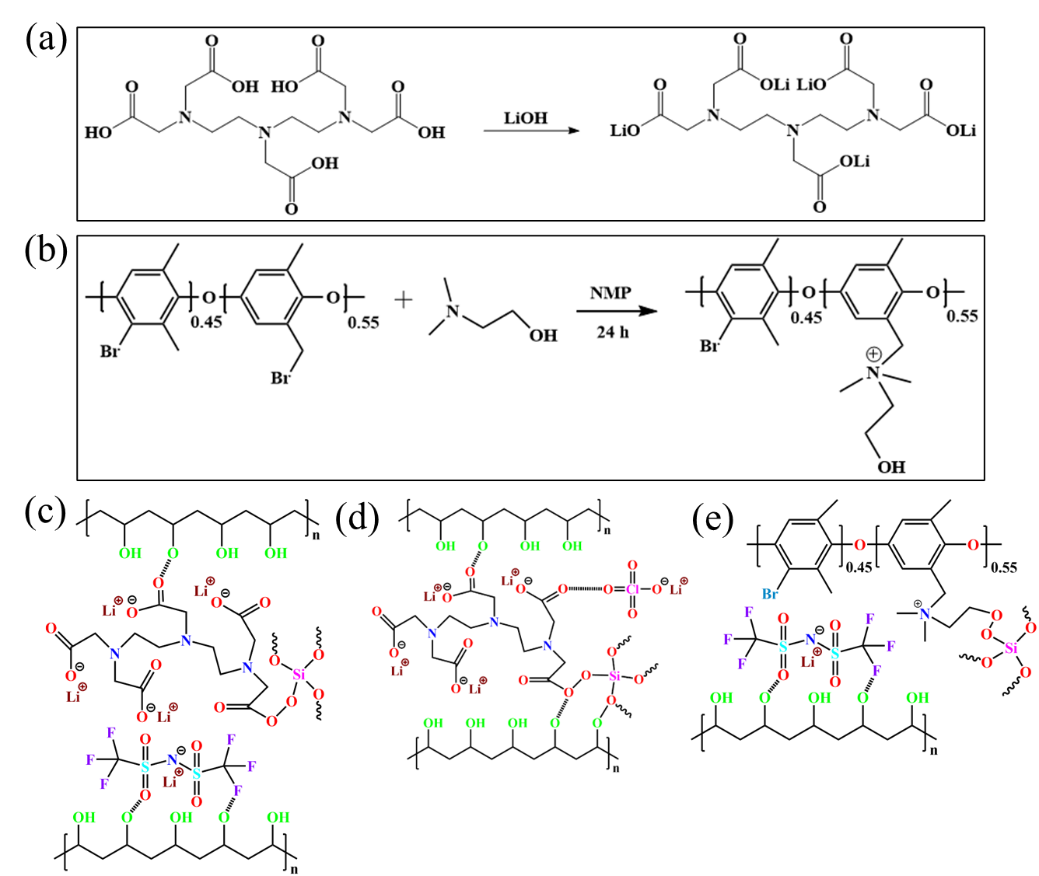


**Figure S1.** Synthesis of (a) Q-DETP, and (b) Q-BPPO, the structural aspects of (c) PDA-F, (d) PDA-Cl, and (e) QBP-F CSPEs.

Preparation of PVA solution (PVA): 5 g of PVA was added to 95 mL of DMSO for 8 h at 95 °C. Afterward, the solution mixture was stirred continuously to prepare a 5% clear solution of PVA.

Synthesis of ion-polarized diethylenetriaminepentaacetic acid (P-DETP): Diethylenetriaminepentaacetic acid of 8 g (20.3381 mmol) was dissolved in 160 mL DMAc and stirred well. Then, 0.5 M of lithium hydroxide solution was added dropwise and stirred continuously until the pH reached 8.0. Afterward, the solution was purified through dialyzed against DMAc/H_2_O (8:2) for 24 h and put in a 60 °C vacuum oven to dry for 24 h to obtain the resultant product **(Figure S1a)**.

Synthesis of quaternized brominated poly (2,6-dimethyl-1,4-phenyleneoxide) (Q-BPPO): 1 g (2.613 mmol) of BPPO was dissolved in NMP to produce 10 % of BPPO solution. Afterward, the dimethylethanolamine was added to the BPPO solution and stirred for 24 h at room temperature. After that, the resultant mixture was precipitated in toluene and the precipitate was obtained using the filtration process. Then, the precipitate was washed with ethyl acetate, and diethyl ether respectively. Afterward, the resultant precipitate was filtered and dried at 40 °C for 48 h in the oven to obtain the Q-BPPO **(Figure S1b)**.

**Table S1.** Compositions of the as-prepared SPEs

| CSPEs | PVA (mL) | LiTFSI (mmol) | LiClO_4_ (mmol) | P-DETP (mmol) | Q-BPPO (mmol) | TEOS (mmol) |
| --- | --- | --- | --- | --- | --- | --- |
| PDA-F | 10 | 6.50 | - | 0.50 | - | 0.11 |
| PDA-Cl | 10 | - | 6.50 | 0.50 | - | 0.11 |
| QBP-F | 10 | 6.50 | - | - | 0.50 | 0.11 |

The conducting solid polymer electrolytes (CSPEs) were synthesized by polymer blending reaction and cross-linking strategy. The 5% PVA solution was initially prepared as the network skeleton of the electrolyte structure, and then the organic macromolecules containing functional group structure were added to the design and synthesis for blending reaction. After complete dissolution, the suitable lithium salt, such as lithium bis((trifluoromethyl)sulfonyl)azanide (LiTFSI) or lithium perchlorate (LiClO_4_), was added and reacted at Ar atmosphere and 60 °C for 24 h. The composition of PVA, P-DETP **(Figure S1a)**, Q-BPPO **(Figure S1b)**, LiTFSI, LiClO_4_, and TEOS were added based on **Table S1**, while the corresponding CSPEs were represented as PDA-F, PDA-Cl, and QBP-F, respectively **(Figure S1c-e)**.

1.3. Materials Characterization

The morphology and element distribution of the CSPEs was observed by scanning electron microscopy (SEM, SU8020, HITA-CHI) and energy-dispersive X-ray spectroscopy (EDS). X-ray photoelectron spectroscopy (XPS, ESCALAB250Xi, America Thermo) was used to analyze the chemical composition of materials. Fourier transform infrared spectroscopy (FTIR) was carried out on a Thermo Nicolet iS50. The thermogravimetric analysis (TGA) was performed on a thermogravimetric analyzer (TGA8000, America PE). A stretching machine (Instron 5965) was used to measure the mechanical properties of materials. The X-ray diffraction (XRD) pattern was taken on a PANalytical X-Pert PRO MPD diffractometer.

1.4. Electrochemical Measurements

The ionic conductivity of CSPEs was calculated by using Eq. (1).^[1]^

$\sigma=\frac{L}{R\cdot S}$ (1)

where L and S are the thickness and surface area of the CSPEs respectively, and R is the bulk resistance obtained by AC impedance assembled with SS|CSPEs|SS batteries, where SS is stainless steel, and the tests were performed from 1 MHz to 0.1 Hz on a CHI760D electrochemical workstation.

Lithium transference number (t_Li+_) was determined on the Li|CSPEs|Li symmetric batteries by combining AC impedance and DC polarization method followed Eq. (2).^[1]^

$t_{Li+}=\frac{I_{s}\left( \Delta V-I_{0}\cdot R_{0} \right)}{I_{0}\left( \Delta V-I_{s}\cdot R_{s} \right)}$ (2)

where, I_s_ and I_o_ are the steady-state current and initial current, respectively. R_s_ and R_o_ are the steady-state resistance and bulk ohmic resistance correspondingly. The impedance spectra were acquired from 1 MHz to 0.1 Hz while the DC polarization with a polarization voltage (ΔV) of 0.01 V.

Li|CSPEs|SS batteries were assembled to measure the electrochemical stabilization window of the electrodes. For the linear sweep voltammetry (LSV) test, the voltage range is between 2.0 and 6.0 V vs. Li/Li^+^ and the scan rate is 10 mV s^-1^.

The LiFePO_4_, conductive carbon, and PVDF were uniformly mixed in NMP at the mass ratio of 8:1:1 to obtain the LiFePO_4_ cathode. Afterward, the slurry was cast on an aluminum foil and dried in a blast drying oven at 80 °C for 24 h. Then, the cathode sheet was cut to a diameter of 16 mm, the active material (LiFePO_4_) was loaded with about 1.2 mg cm^-2^, and the cathode thickness was ~75 μm. The LiNi_0.88_Co_0.06_Mn_0.06_O_2_ (NCM8866) and LiNi_0.90_Co_0.05_Mn_0.05_O_2_ (NCM9055) were prepared by the same method as above, but LiFePO_4_ was replaced by NCM8866 or NCM9055. For electrochemical measurements, the cathode, electrolyte film, and lithium anode were assembled into a half cell (CR2032) in the glove box with Ar atmosphere (O_2_ < 0.5 ppm, H_2_O < 0.5 ppm). The charge/discharge cycling was carried out over the voltage range of 2.8-3.6 V (LiFePO_4_ batteries), 2.8-4.2 V (NCM8866 batteries) or 2.8-4.3 V (NCM9055 batteries) using the Neware battery testing system (Shenzhen Neware Electronic, China). The CV tests were performed at a scanning speed of 10 mV s^-1^ in LiFePO_4_ cells from 2.5 to 4.2 V.

2. Results and Discussion


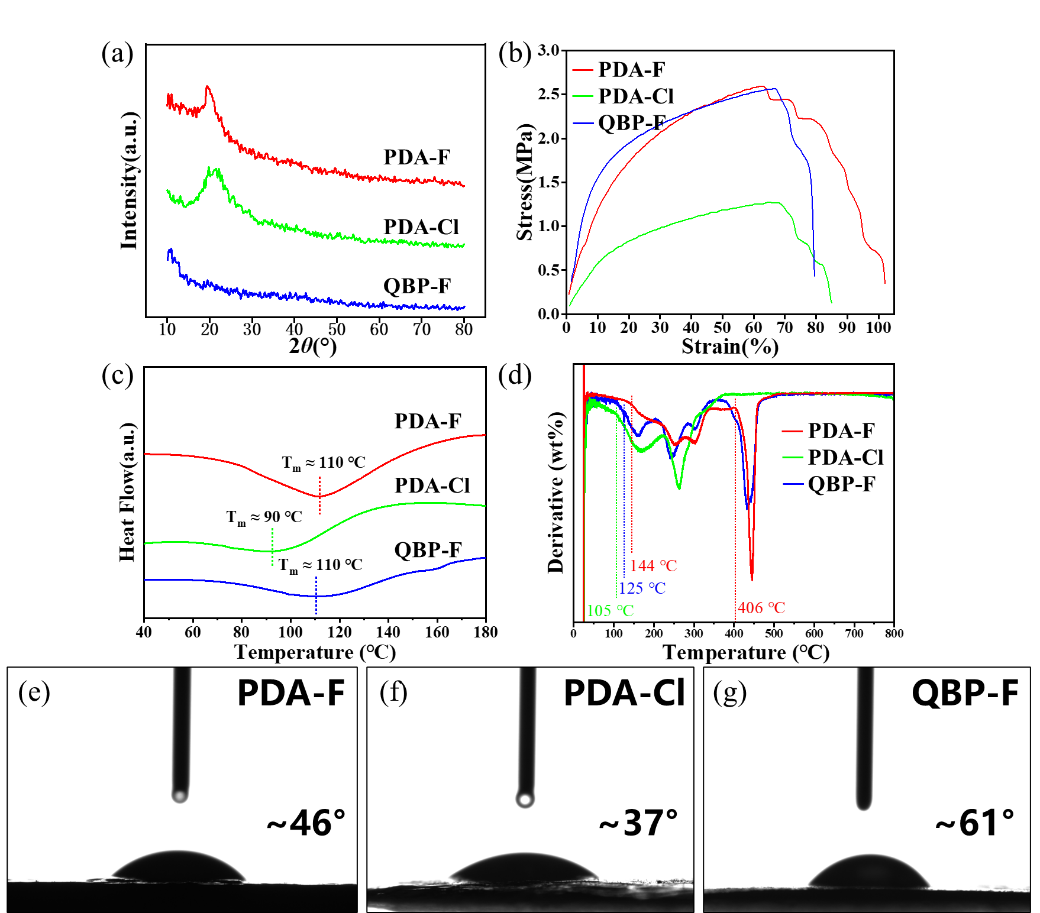


Figure S2. (a) XRD spectra of CSPEs. (b) Stress-strain curves of CSPEs. (c) DSC profiles of CSPEs. (d) DTGA thermograms of CSPEs. The water contact angle of (e) PDA-F, (f) PDA-Cl, and (g) QBP-F.

As shown in **Figure S2a**, the various samples present only one broad peak at about 2θ = 19.6° in the XRD patterns, suggesting the amorphous structure for all membrane samples.^[2]^ There are various interactions between the quaternary ammonium macromolecular spacer matrix and lithium salts, which reduce the crystallinity of the electrolyte membrane and improve the ionic conductivity. To evaluate the mechanical properties of CSPEs, we performed stress-strain tests, as shown in **Figure S2b**. In order to assess the safety of the battery during charging and discharging, we conducted thermal stability tests on the CSPEs **(Figure S2c-d)**. The melting temperature of the electrolyte film was determined through differential scanning calorimeter (DSC) tests at 30-200 °C. As depicted in **Figure S2c**, PDA-Cl exhibits a peak heat absorption at 90 °C, while PDA-F and QBP-F demonstrate melting points of approximately 110 °C. Furthermore, thermogravimetric analysis (TGA) was performed on CSPEs which revealed that they initially experienced slight weight loss primarily due to water evaporation as a result of varying degrees of water absorption from air **(Figure S2d)**. Subsequently, with increasing temperature, mass continues to decrease attributable to residual DMSO volatilization. PDA-Cl reaches decomposition temperature at ~225 °C and undergo significant weight loss; QBP-F begin decomposing at ~375 °C; and PDA-F exhibits a decomposition temperature reaching ~406 °C. The impressive thermal stability displayed by PDA-F may be attributed to enhanced polymer network density and robustness resulting from hydrogen bond interactions between amine polymeric polymer and PVA chain, effectively improving film's mechanical properties. The CSPEs were cut into strips 20 mm long and 10 mm wide. Test them with a pull machine. The results show that the tensile strength of PDA-F, PDA-Cl, and QBP-F are respectively 1.95 MPa, 1.28 MPa, and 1.16 MPa. Their fracture nominal strains are 102.1%, 84.9%, and 79.4%. To verify the hydrophilic-hydrophobicity of CSPEs, we have performed the water contact angle tests (**Figure S2e-g**). Both macromolecular spacers and lithium salts can affect the hydrophilicity of the electrolyte membrane. The hydrophobicity of PDA-F, PDA-Cl and QBP-F are respectively 46°, 37°and 61°. The improvement of the hydrophobicity of the electrolyte membrane could reduce the influence of ambient air to a certain extent, which is conducive to increasing the mobility of lithium ions inside the membrane. Hence, it is beneficial to improve the cycle performance of the batteries.


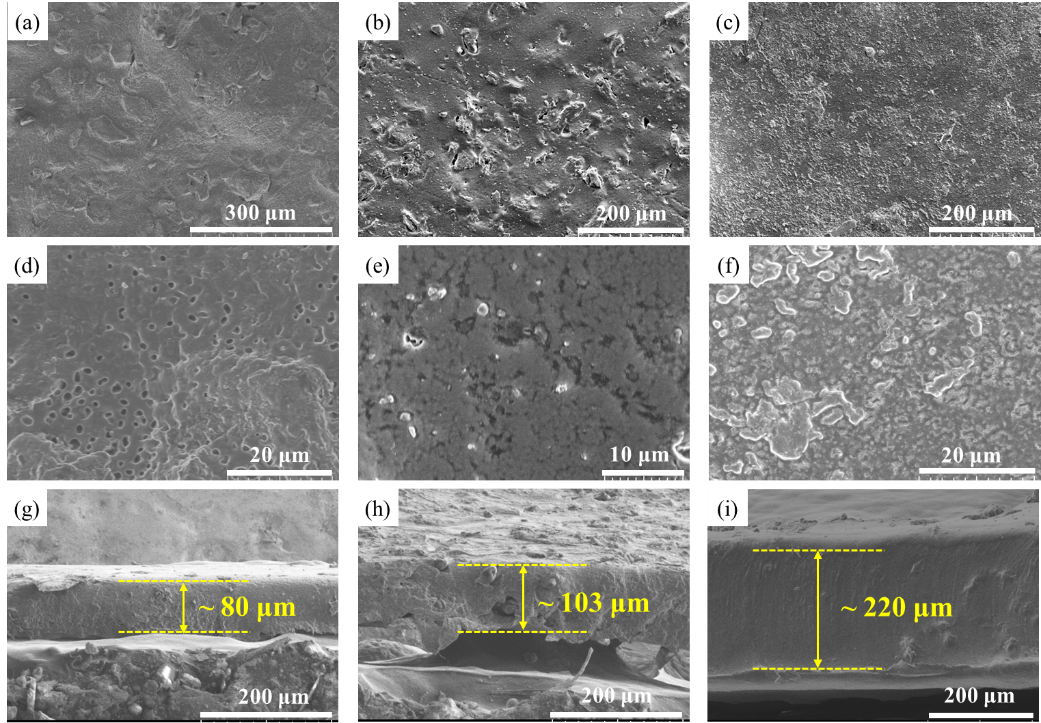


**Figure S3.** Surface SEM images of (a, d) PDA-F CSPE. (b, e) PDA-Cl CSPE. and (c, f) QBP-F CSPE. cross-sectional SEM images of (g) PDA-F CSPE. (h) PDA-Cl CSPE. (i) QBP-F CSPE.

**
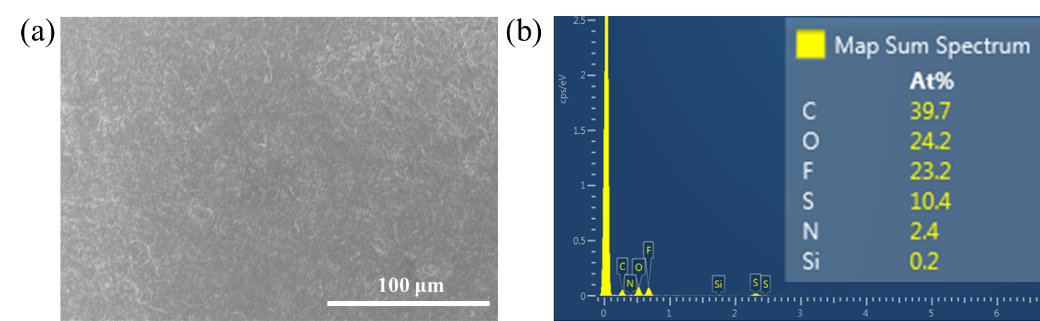
**

**Figure S4.** (a) SEM image of the surface at 100 μm of PDA-F CSPE. (b) EDS spectrum of PDA-F CSPE.

To study the interfacial properties of the electrolyte membranes, we performed SEM and EDS tests on them (**Figure S3, S4**). The smooth and homogeneous surface of the membrane facilitates good interface contact with the electrodes, resulting in better SEI. Compared to other CSPEs, PDA-F has the smoothest surface. However, the PDA-Cl has a rough morphology, while the surface of QBP-F is uneven, which is not conducive to good interface contact. Poor interfacial contact will lead to an increase in interface impedance, thereby reducing the conductivity of the electrolyte membrane, which is not conducive to the exertion of battery capacity density. Considering the EDS results, the surface of PDA-F has C, F, O, S, N, and Si elements (**Figure S4**). Since the skeleton of the electrolyte membrane is a carbon chain, it is rich in carbon. The PVA matrix consists of numerous hydroxyl groups and provides a large amount of -OH. LiTFSI imparts F, but also O and S, while Q-DETP provides N and O, and Si comes from tetraethyl orthosilicate. The thickness of CSPEs has been made as thin as possible. If it is too thin, the mechanical properties of the film will be very poor and it will be hard to remove from the glass plate. In addition, the polymer self-supporting film that is too thin has poor resistance to lithium dendrites, which can easily make the batteries short-circuit and failed during operation.


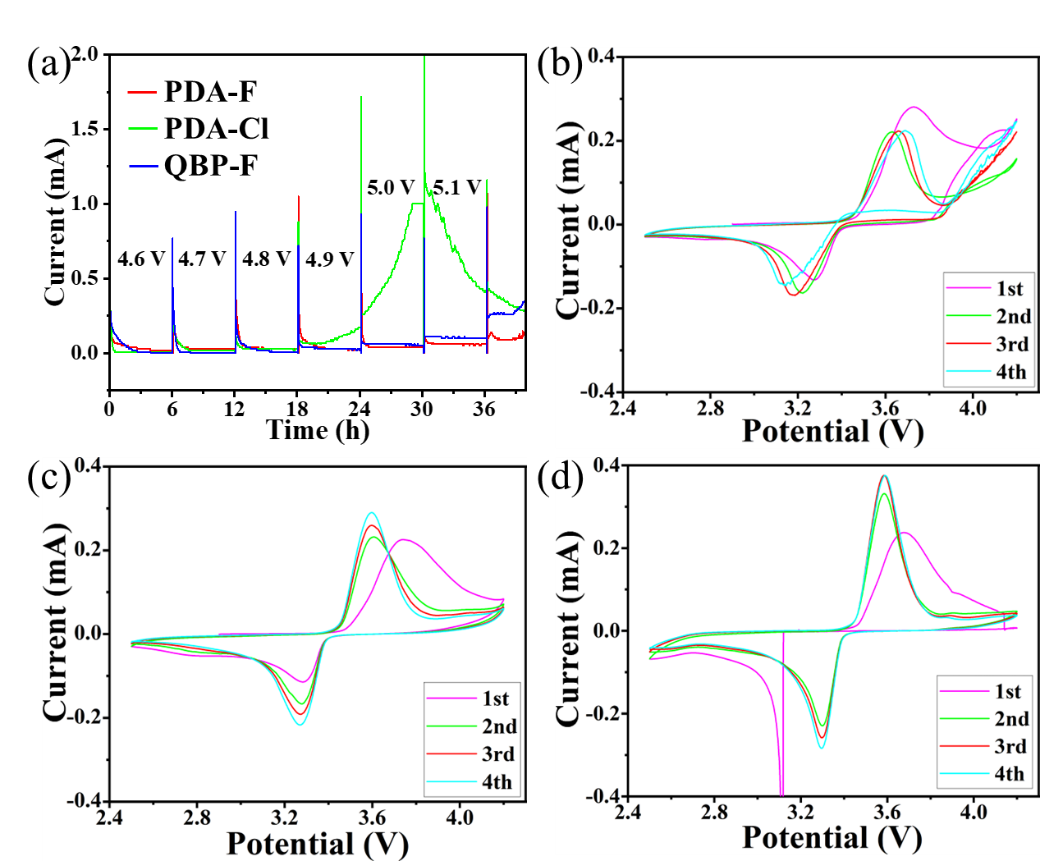


**Figure S5.** (a) Electrochemical floating analysis of CSPEs. CV curves of the LFP/CSPEs/Li cells with (b) PDA-F, (c) PDA-Cl, and (d) QBP-F between +2.5 and +4.2 V (vs. Li/Li^+^) at 25 °C and 0.1 mV s^-1^ scan rate.

The measured leakage current can directly evaluate the actual oxidation stability of the electrolyte. The results are shown in **Figure S5a**. As can be seen from **Figure S5a**, the leakage current of PDA-F is small and stable when it is below 5.1 V, and increases sharply when it is 5.2 V, indicating that its electrochemical window is ~ 5.1 V, which fully proves its excellent stability to high voltage cathodes. Similarly, the electrochemical windows for PDA-Cl and QBP-F are ~ 4.8 V and ~ 5.0 V, respectively. LiFePO_4_/CSPEs/Li cells were assembled for cyclic voltammetry (CV) testing to further investigate the electrochemical stability of the membranes (**Figure S5b-d**). The CV curves are tested in the voltage range of +2.5 ~ 4.2 V (vs. Li/ Li^+^) at 25 °C and 0.1 mV s^-1^ scan rate to obtain 4 cycle data. It can be seen that each CSPE will undergo an irreversible phase transition in the first cycle due to the interface contact problem. The curves in the last three circles are similar in shape. The results showed that P-DETP, Q-BPPO, and TEOS had no side reactions. The reversible redox peaks are mainly formed by the intercalation and separation of Li+ in the electrode. LiFePO_4_/QBP-F/Li has the smallest curved integral area, indicating that P-DETP is more conducive to improving battery capacity than Q-BPPO.


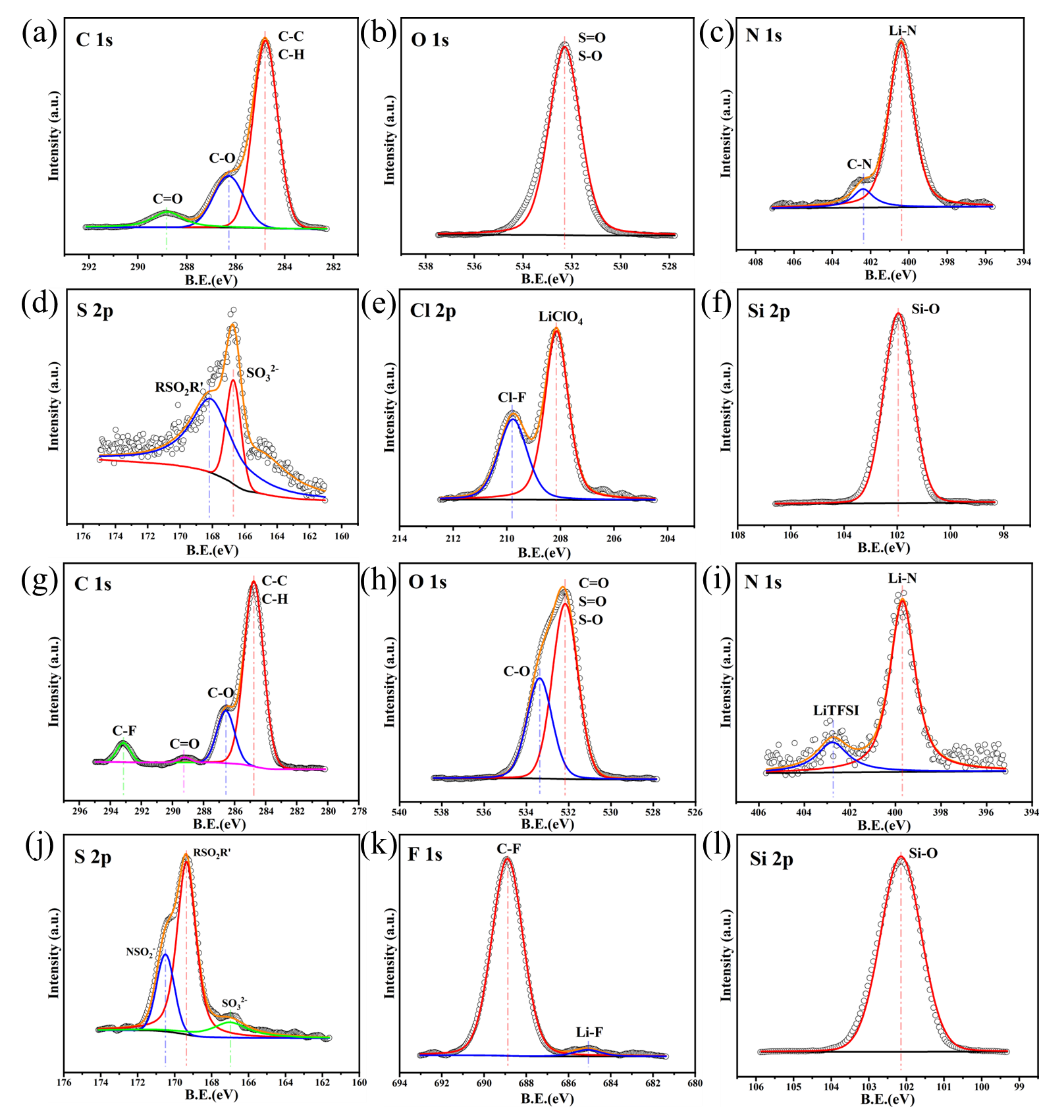


**Figure S6.** (a-f) The high-resolution C 1s, O 1s, N 1s S 2p, Cl 2p, and Si 2p of PDA-Cl CSPE, (g-l) The high-resolution C 1s, O 1s, N 1s S 2p, F 1s, and Si 2p of QBP-F CSPE.

**Figure S6** is the XPS analysis plot of PDA-Cl and QBP-F CSPEs. The high-resolution XPS survey demonstrates the occurrence of the predictable elements C 1s, O 1s, N 1s, S 2s, F 1s, Cl 2p, and Si 2p. As a polymer matrix framework, PVA provides a large number of C-C bonds and C-O ether bonds **(Figure S6a-b, S6g-h)**. Based on this, the −OH groups of PVA play a hydrogen bonding role to provide a passage for the directional movement of lithium ions, and due to the limited size of this channel, it could prevent the entry of anions with larger diameters, thereby effectively separating Li^+^ ions and anions.^[3]^ To effectively limit the movement of lithium salt anions and release more free lithium ions. It is beneficial to improve ionic conductivity and lithium-ion migration. Studies have shown that Li_3_N is one of the main components of SEI that inhibits the growth of lithium dendrites and can be stable in contact with the lithium anode interface.^[4]^ **Figure S6c** and **S6i** show that CSPEs successfully form Li-N structure. Furthermore, LiF in the SEI can also inhibit the formation of lithium dendrites, therefore, LiF is also one of the important components in the SEI.^[5]^ Lithium halides usually have no side reaction with lithium metal and have good chemical stability. In addition, lithium halide typically has a relatively large band gap (LiF: 8.70 eV and LiCl: 7.62 eV),^[6]^ which allows them to be isolated on the side of Li anode through possible electron transport, like electrical insulators. According to previous studies,^[7]^ lithium halides play significant roles in resisting lithium dendrites and inhibiting the occurrence of interface side reactions. **Figure S6e** and **S6k** confirmed the formation of lithium halide. In addition, the incorporation of tetraethyl orthosilicate helps to form a Si-O structure **(Figure S6f, S6l)**, which can promote the dissociation of lithium salts through electrostatic interaction, which is conducive to the formation of a three-dimensional conductive network with rapid transport of lithium ions.


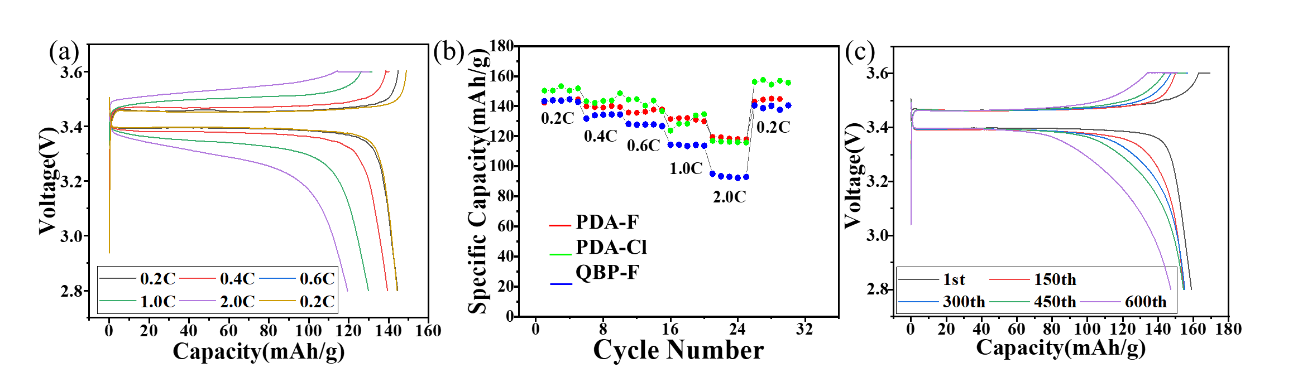


**Figure S7.** (a) Galvanostatic charge/discharge profiles of the LFP/PDA-F/Li cell at 25 °C at 0.2-2 C rates. (b)The rate capability of LFP/CSPEs/Li cells for PDA-F, PDA-Cl, and QBP-F CSPEs at 0.2-2 C rates. (c) Voltage plateaus of the 1st, 150th, 300th, 450th, and 600th cycles of the LFP/PDA-F/Li battery at 0.3 C and 25 °C.

Considering these analyses, it can be seen that PDA-F performed well. It is assembled into a coin cell battery with an LFP anode and a lithium metal cathode and examined the rate and cycle performance of the batteries as well **(Figure S7)**. The performance of the batteries at different magnifications was observed in the voltage range of 2.8-3.6 V. The LFP/PDA-F/Li cell could deliver the specific discharge capacity of 147.9 mAh g^-1^, 138.4 mAh g^-1^, 136.3 mAh g^-1^, 128.1 mAh g^-1^, and 112.9 mAh g^-1^ at 0.2 C, 0.4 C, 0.6 C, 1.0 C, and 2.0 C respectively **(Figure S7b)**. After that, at a rate of 0.2 C, the discharge capacity of the battery can still be maintained at 151.3 mAh g^-1^, showing the long cycle performance. Then, the assembled LFP/PDA-F/Li cell is tested for long-cycle performance considering the voltage range of 2.8-3.6 V at 25 °C and 0.3 C. It can export a high initial specific discharge capacity of 153.5 mAh g^-1^ with an acceptable cycling performance and retains 146.7 mAh g^-1^ after 600 cycles with a capacity retention of 95.57%.

**
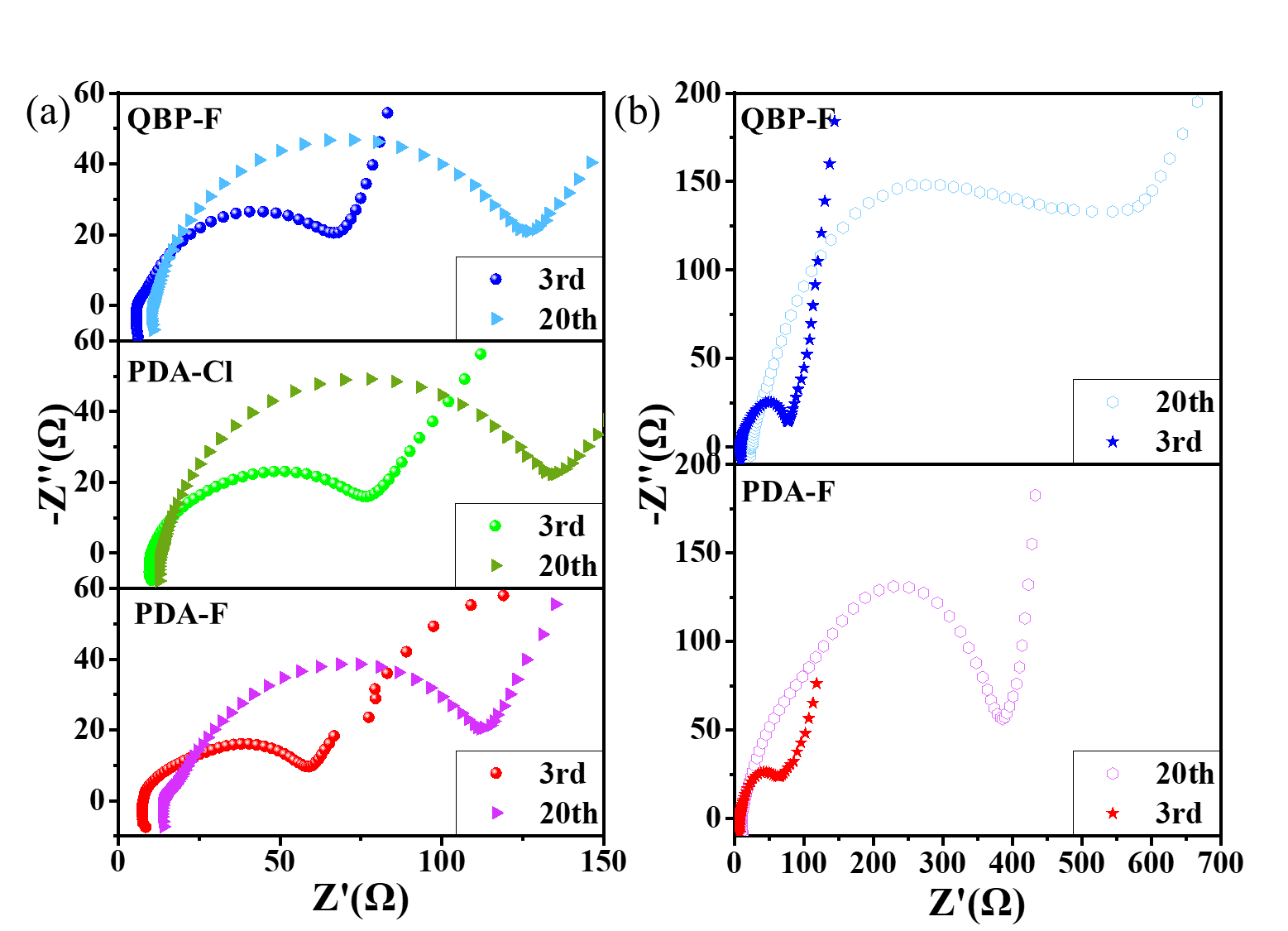
**

**Figure S8.** (a) EIS spectra of LFP/Li batteries after 3rd and 20th cycles at 0.5 C. (b) EIS spectra of NCM8866/Li batteries after 3rd and 20th cycles at 0.5 C.

In order to better understand and analyze the internal resistance changes of the battery during charge and discharge, the charge transfer resistance (R_ct_) of the LFP/Li battery and the NCM8866/Li battery were tested respectively **(Figure S8)**. The Nyquist impedance diagram of the cell after the 3rd and 20th cycles at 0.5 C consists of two semicircles and a diachronic line. After 3 cycles, the first smaller semicircle in the high frequency region is attributed to the SEI layer resistance. The SEI resistance **(Figure S8a)** of the LFP/Li cell sample cycled with PDA-F electrolyte (51.84 Ω) is relatively lower than that of the sample cycled with PDA-Cl electrolyte (66.6 Ω) and QBD-F (60.73 Ω). The semicircle in the mid-frequency region represents the charge transfer resistance (R_ct_). Compared to the other two, the R_ct_ of LFP/PDA-F/Li only increased to 97.9 Ω, with minimal change in internal resistance **(Figure S8a)**. In contrast, the R_ct_ of the NCM8866/PDA-F/Li increased from 56.77 Ω to 375.3 Ω. Even, the R_ct_ of NCM8866/QBD-F/Li increased sharply from 68.83 Ω to 520.4 Ω **(Figure S8b)**. The small internal resistance variation and high oxidation-reduction kinetics of PDA-F provide the basis for the construction of cyclically stable LIBs at high current density. The results show that the internal resistance of PDA-F in LFP cell is much smaller than that in NCM8866 cell. The small internal resistance change of LFP/PD-F/Li battery indicates that the interface stability of electrolyte is good and provides favorable conditions for the construction of cyclically stable LIBs. However, NCM8866/PDA-F/Li has large internal resistance, indicating that its electrolyte interface is poor, resulting in a decrease in battery capacity.


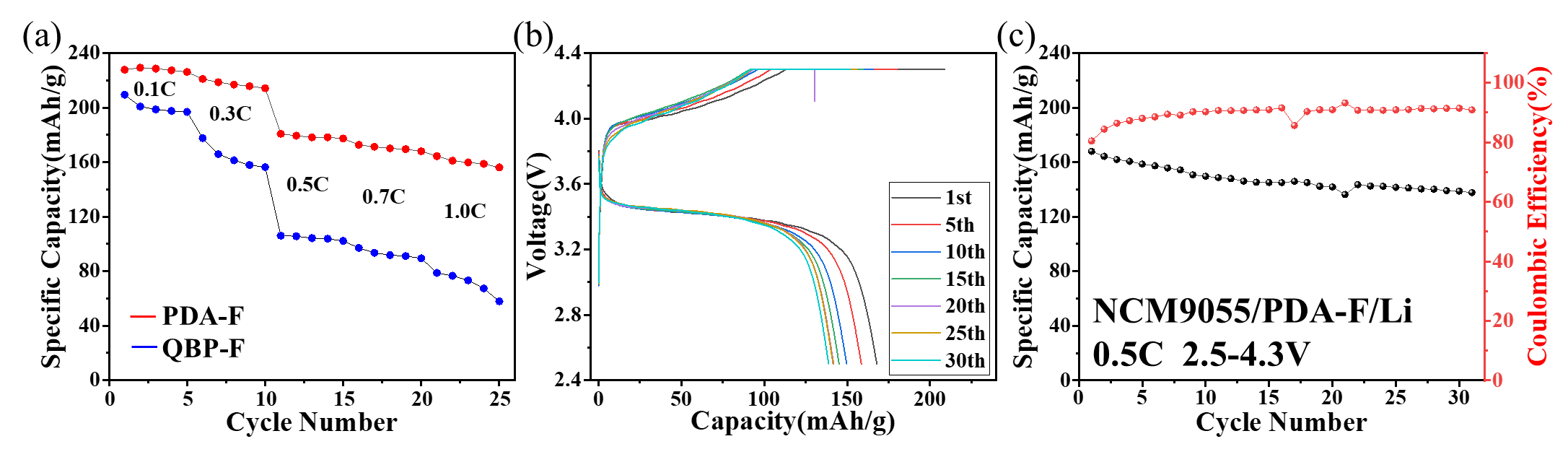


**Figure S9.** (a) The rate capability of NCM9055/CSPEs/Li cells for PDA-F, and QBP-F CSPEs at 0.1-1 C rates. (b) Voltage plateaus of the 1st, 5th, 15th, 20th, 25th, and 30th cycles of the NCM9055/PDA-F/Li battery. (c) Discharge capacity and coulombic efficiency of NCM9055/PDA-F/Li cell at 0.5 C.

Based on the above research, we further assembled NCM9055/Li cells to evaluate the performance and application of PDA-F and QBD-F electrolytes. The NCM9055/PDA-F/Li cell delivers outstanding rate performance with discharge capacities of 226.12, 214.27, 177.41, 167.99, and 156.13 mAh g^-1^ at rates of 0.1, 0.3, 0.5, 0.7, and 1.0 C, respectively **(Figure S9a)**. However, the rate performance of the NCM9055/QBD-F/Li cell is significantly worse. The NCM9055/PDA-F/Li cell is tested for cycling performance in a voltage range of 2.5-4.3 V **(Figure S9b-c)**. Its initial discharge capacity at 0.5 C is 167.94 mAh g^-1^. After 31 cycles at 0.5 C, the discharge capacity is 137.66 mAh g^-1^ and the capacity retention rate is 81.97%. The coulombic efficiency of the battery in the working process is maintained at about 90%.

**Table S2.** Comparison of electrochemical properties of various CSPEs materials.

| LFP/CSPEs/Li | σ/ (S cm^-1^) | LTN | Performance  (mAh g^-1^) | | Ref. |
| --- | --- | --- | --- | --- | --- |
| LFP/PDA-F/Li | 2.8×10^-4^ | 0.869 | 147.9 (0.2 C) | This work | |
| LFP/IBD_30_-PEO/Li | 5.48×10^-4^ (60 °C) | 0.43 | 134 (0.2 C) | [8] | |
| LFP/PMPyrr/Li | 1.4×10^-4^ | 0.71 | 137(0.1 C) | [9] | |
| LFP/PIM-CONH_2_/BC-3h/Li | 3.31×10^-4^ | 0.76 | 140 (0.2 C) | [10] | |
| LFP/DB-SHPE-5/Li | 4.65×10^-5^ (30 °C) | 0.63 | 140 (0.2 C) (60 °C) | [11] | |
| LFP/2PEO-PECP-15/Li | 3.54×10^-5^ | 0.23 | 133 (0.1 C) (40 °C) | [12] | |
| LFP/CPE-4/Li | 2.05×10^-5^ | 0.11 | 150 (0.05 C) (75 °C) | [13] | |
| LFP/7.5%P@SiO_2_@P-PEO-1/8LiClO_4_/Li | 1.3×10^-4^ | 0.81 | 139.4 (0.2 C) | [14] | |

Data not indicated are measured at room temperature (25 °C).

**References**

[1] Y. Zhang, M. Irfan, Z. Yang, K. Liu, J. Su, W. Zhang, *Chem. Eng. J.* **2022**, *435*, 134775.

[2] X. Song, Y. Zhang, Y. Ye, Z. Liu, F. Cheng, H. Li, *ACS Appl. Energy Mater.* **2020**, *3*, 4906-4913.

[3] a) J. Atik, D. Diddens, J. H. Thienenkamp, G. Brunklaus, M. Winter, E. Paillard, *Angew. Chem. Int. Ed.* **2021**, *60*, 11919-11927; b) P. Ding, Z. Lin, X. Guo, L. Wu, Y. Wang, H. Guo, L. Li, H. Yu, *Mater. Today* **2021**, *51*, 449-474; c) D. Lin, Y. Liu, Y. Cui, *Nat. Nanotechnol.* **2017**, *12*, 194-206.

[4] a) Y. Ren, S. Yang, X. Ma, Z. Qi, C. Zhang, X. Liu, X. Tan, S. Sun, *Mater. Today Chem.* **2021**, *21*, 100510; b) S. Liu, X. Xia, S. Deng, D. Xie, Z. Yao, L. Zhang, S. Zhang, X. Wang, J. Tu, *Adv. Mater.* **2019**, *31*, 1806470.

[5] a) J. Tan, J. Matz, P. Dong, J. Shen, M. Ye, *Adv. Energy Mater.* **2021**, *11*, 2100046; b) Y. Yuan, F. Wu, Y. Bai, Y. Li, G. Chen, Z. Wang, C. Wu, *Energy Storage Mater.* **2019**, *16*, 411-418; c) X. Yang, M. Jiang, X. Gao, D. Bao, Q. Sun, N. Holmes, H. Duan, S. Mukherjee, K. Adair, C. Zhao, J. Liang, W. Li, J. Li, Y. Liu, H. Huang, L. Zhang, S. Lu, Q. Lu, R. Li, C. V. Singh, X. Sun, *Energy Environ. Sci.* **2020**, *13*, 1318-1325; d) Y. Liu, R. Hu, D. Zhang, J. Liu, F. Liu, J. Cui, Z. Lin, J. Wu, M. Zhu, *Adv. Mater.* **2021**, *33*, 2004711; e) C. Gong, S. D. Pu, X. Gao, S. Yang, J. Liu, Z. Ning, G. J. Rees, I. Capone, L. Pi, B. Liu, G. O. Hartley, J. Fawdon, J. Luo, M. Pasta, C. R. M. Grovenor, P. G. Bruce, A. W. Robertson, *Adv. Energy Mater.* **2021**, *11*, 2003118.

[6] M. Shahrokhi, B. Mortazavi, *Comput. Mater. Sci* **2018**, *143*, 103-111.

[7] a) K. Zhang, F. Wu, K. Zhang, S. Weng, X. Wang, M. Gao, Y. Sun, D. Cao, Y. Bai, H. Xu, X. Wang, C. Wu, *Energy Storage Mater.* **2021**, *41*, 485-494; b) J. Wang, Z. Zhang, H. Ying, G. Han, W.-Q. Han, *Chem. Eng. J.* **2021**, *411*, 128534.

[8] S. Wang, J. Li, T. Li, W. Huang, L. Wang, S. Tao, *Chem. Eng. J.* **2023**, *461*, 141995.

[9] J. C. Barbosa, R. S. Pinto, D. M. Correia, C. R. Tubio, R. Gonçalves, C. M. Costa, S. Lanceros-Mendez, *J. Power Sources* **2023**, *585*, 233630.

[10] H. Zou, Y. Wang, X. Li, P. Ding, H. Guo, F. Li, *J. Membr. Sci.* **2023**, *685*, 121939.

[11] B. Zhou, T. Deng, C. Yang, M. Wang, H. Yan, Z. Yang, Z. Wang, Z. Xue, *Adv. Funct. Mater.* **2023**, *33*, 2212005.

[12] B. Huang, P. Lai, H. Hua, R. Li, X. Shen, X. Yang, P. Zhang, J. Zhao, *J. Power Sources* **2023**, *570*, 233049.

[13] X. Cai, Z. Cai, H. Yuan, W. Zhang, S. Wang, H. Wang, J. Lan, Y. Yu, X. Yang, *J. Colloid Interface Sci.* **2023**, *648*, 972-982.

[14] Z. Chen, H. Jia, S. Yan, J.-F. Gohy, *Nano Energy* **2023**, *114*, 108637.
